# Meibomian gland secretion quality association with ocular parameters in university students during COVID- 19 restrictions

**DOI:** 10.1007/s10792-023-02632-2

**Published:** 2023-01-24

**Authors:** Jacobo Garcia-Queiruga, Hugo Pena-Verdeal, Belén Sabucedo-Villamarin, Maria J. Giraldez, Carlos Garcia-Resua, Eva Yebra-Pimentel

**Affiliations:** GI-2092 - Optometry, Department of Applied Physics (Optometry Area), Universidade de Santiago de Compostela, Campus Vida s/n, Santiago de Compostela, Spain

**Keywords:** Meibomian gland secretion quality, Meibomian gland orifice plugging, Video display terminals, Eyelid abnormalities, Meibomian gland loss area

## Abstract

**Purpose:**

To determine if the Meibomian Gland (MG) secretion quality is associated with symptoms of ocular discomfort, hours of Video Display Terminals (VDT) use, eyelid margin abnormalities, conjunctival hyperemia, and Meibomian Gland Loss Area (MGLA) in a sample of university students.

**Methods:**

An online survey that included an Ocular Surface Disease Index (OSDI) questionnaire and an extra question about hours of VDT use recruited an initial sample of 183 participants. Only 120 participants that fulfilled the inclusion criteria were scheduled for a battery of ocular surface and MG specific exam. The tests include: 1) meibometry, 2) slit lamp exploration of eyelid margin abnormalities (irregularity, hyperemia and MG orifices plugging), MG secretion quality and conjunctival hyperemia, and 3) Meibography.

**Results:**

Significant positive correlations between the MG secretion quality and eyelid margin hyperemia, MG orifices plugging, MGLA, nasal conjunctival hyperemia, and temporal conjunctival hyperemia (Spearman Rho; all r>0.186, p<0.042) were found. Multivariate regression found association between OSDI with hours of VDT use (B=0.316, p=0.007), and eyelid hyperemia (B=0.434, p≤ 0.001). A statistical association between MG secretion quality and eyelid margin hyperemia, MG orifices plugging, MGLA and conjunctival hyperemia (Fisher’s exact; all p<0.039) were found. Multivariate regression found association between MG secretion quality with MG orifices plugging (B=0.295, p=0.004) and meibometry (B=-0.001, p=0.029).

**Conclusion:**

Participants with higher values in MG secretion quality have higher values in eyelid margin hyperemia, MG plugging, MGLA, and conjunctival hyperemia. No direct relationship between MG secretion quality and hours of VDT use or OSDI were found.

## Introduction

The coronavirus disease 2019 (COVID-19) pandemic has caused many lifestyle changes in the worldwide population [1, 2]. In particular, university students were affected in the way they received their lessons, which were telematic during the lockdowns [3]. The number of hours using Video Display Terminals (VDT) by university students has risen from 4 h to more than 8 h per day due to limitations on face-to-face lessons [2]. Ocular discomfort is highly correlated with the hours of VDT use because of the reduction of complete blinks, which are important to maintain the ocular surface homeostasis [4–7]. When a complete blink occurs, the apposition of the lower and the upper eyelid borders spread the tear film lipid layer all over the ocular surface [8]. The Meibomian Glands (MG) are located in the superior and inferior tarsal plate along the upper and lower eyelids with their orifices along the free eyelid margins [8]. These sebaceous glands produce the meibum, the main component of the tear film lipid layer, which plays an important role in avoiding tear film evaporation and maintaining a smooth optical surface [8, 9]. Incomplete blinking reduces the meibum spreading over the ocular surface, contributes to an accumulation of fatty acids over the eyelids free border, and provokes obstruction of the MG orifices with the subsequent reduction of the MG secretion quality [10, 11]. Therefore, the aim of the present study was to determine if the quality of the MG secretion is associated with symptoms of ocular discomfort, hours of VDT use, eyelid margin abnormalities, conjunctival hyperemia, and Meibomian Gland Loss Area (MGLA) in a sample of university students.

## Methods

### Study design

All participants were between 19 and 32 years old and were recruited from students and attendants to the Optometry Faculty to be involved in a cross-sectional study. The present study was adhered to the tents of the Declaration of Helsinki and was approved by the Bioethics Committee of the institution. Informed consent was signed by every participant to be included in the study. All participants who wanted to participate have completed an online form which could be accessed by scanning a QR code. The self-administered survey form was formed by a full Ocular Surface Disease Index (OSDI) questionnaire and an extra question about hours of VDT use (*“How many hou*r*s do you use video elect*r*onic displays du*r*ing a day?”*) [12].

**Fig. 1:**
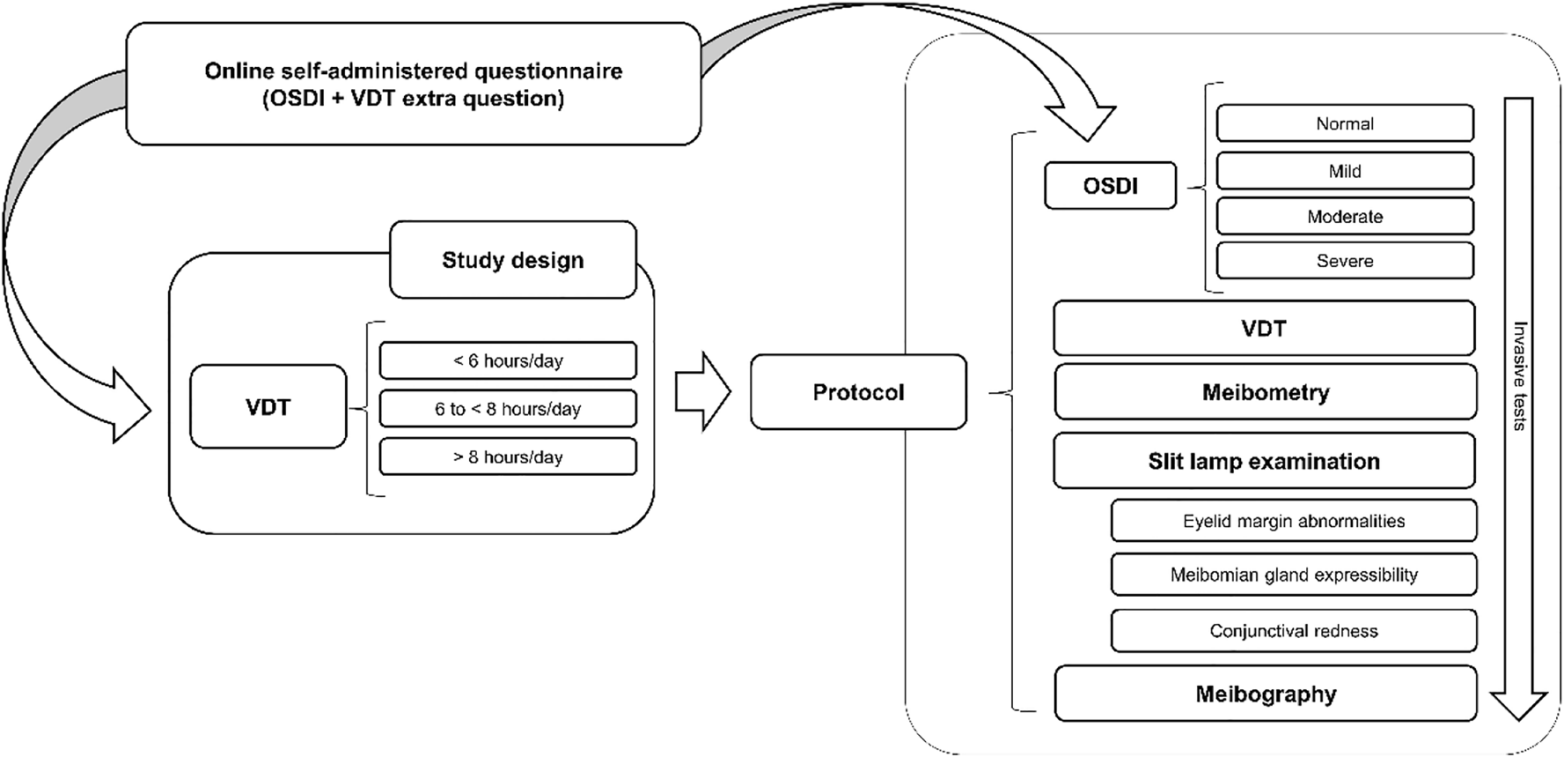
Summary of the study design and protocol session. *OSDI*—ocular surface disease index, *VDT*—video display terminals

A total of 183 volunteers completed the online self-administered survey. Volunteers were scheduled for an eye examination but first underwent an oral history taking to check if they could be included in the study. To be included in the study, participants had to have no history of a conjunctival, scleral, or corneal disease, active ocular disease or ocular allergy, prior eye surgery (including refractive surgery or eyelid tattooing), glaucoma, diabetes mellitus, thyroid disorders, were pregnant or breast-feeding, wore contact lenses, had a systemic inflammatory/autoimmune disease or were following any pharmacological treatment/systemic drug that can disrupt the normal function of the ocular surface. Also, participants had to be between the ages 18 and 35 years old and enrolled in the university before march of 2020. The study design was planned to create 3 groups of 40 participants each (depending on their responses about the VDT extra question): group 1– < 6 h/day; group 2–6 to < 8 h/day; and group 3 – > 8 h/day. The study design and protocol session are summarized in Fig. 1.

120 out of the 183 volunteers (mean ± Standard Deviation [SD] age 22.5 ± 2.32 years) who met the inclusion criteria were scheduled for the study session. All tests were performed in a single appointment. These tests included: meibometry, eyelid margin abnormalities, quality of the MG secretion, conjunctival hyperemia, and meibography were measured. To avoid any possible alteration, less invasive tests were performed first (Fig. 1) [13]. All measurements were performed in the morning from 10.00 a.m. to 1.00 p.m. by the same observer. Data were masked with an alphanumeric code for the analysis by a second experienced observer.

### Ocular discomfort symptomatology and hours of video displays terminals use

Symptomatology of ocular surface discomfort was quantified by an online self-administered OSDI questionnaire [14, 15]. Participants were categorized into 4 severity grades depending on their OSDI values [15]. For the hours of VDT use quantification, participants have completed an extra question in the online self-administered survey and were categorized into three grades. The categorization of OSDI and hours of VDT use are summarized in Table 1.

**Table 1:** Summary of the test procedures, material, method, and categorization performed

Parameter	Material	Method	Categorization
OSDI	OSDI questionnaire	Online self − administered questionnaire. Access to the survey by QR code	Distributed in 4 grades [14, 15]:
			Grade 1 (Normal [0, 12))
			Grade 2 (Mild [12, 22))
			Grade 3 (Moderate [22, 32))
			Grade 4 (Severe [32, 100))
Hours of VDT use	Extra question in online questionnaire *(“How many hou*r*s do you use video elect*r*onic displays du*r*ing a day?”)*	Online self − administered questionnaire. Access to the survey by QR code	Distributed in 3 grades:
			Grade 1 (< 6 h/day)
			Grade 2 (6 to < 8 h/day)
			Grade 3 (> 8 h/day)
Meibometry	Meibometer MB560	Matt synthetic tape in contact with the eyelid margin during 10 s	No categorization stabilized. This parameter follows a linear distribution [18]
Eyelid margin irregularity	Topcon SL − D2 slit lamp with DC4 video recording system attached	1 video was recorded Settings on 16 × and white diffuse light	Arita et al. [20] grading scale:
			Grade 0 (No irregularity)
			Grade 1 (Fewer than 3 irregularities with shallow notching)
			Grade 2 (Three or more irregularities or deep notching)
Eyelid margin hyperemia	Topcon SL-D2 slit lamp with DC4 video recording system attached	1 video was recorded settings on 16 × and white diffuse light	Arita et al. [20] grading scale:
			Grade 0 (No or slight redness and no telangiectasia crossing MG orifices)
			Grade 1 (Redness and no telangiectasia crossing MG orifices)
			Grade 2 (Redness and telangiectasia crossing MG orifices, less than half of the full length of the lid)
			Grade 3 (Redness and telangiectasia crossing MG orifices, half or more of the full length of the lid)
Plugging of the MG orifices	Topcon SL-D2 slit lamp with DC4 video recording system attached	1 video was recorded Settings on 16 × and white diffuse light	Arita et al. [20] grading scale:
			Grade 0 (No plugging)
			Grade 1 (Fewer than 3 pluggings)
			Grade 2 (Three or more pluggings, less than half of the full length of the lid)
			Grade 3 (Three or more pluggings, half or more of the full length of the lid)
Quality of the MG secretion	Topcon SL-D2 slit lamp with DC4 video recording system attached	1 video was recorded Settings on 16 × or 25 × and white diffuse light	Bron et al. [22] quality of meibum grading scale:
			Grade 0 (clear)
			Grade 1 (cloudy)
			Grade 2 (granular)
			Grade 3 (opaque solid/toothpaste)
Conjunctival hyperemia	Topcon SL-D2 slit lamp with DC4 video recording system attached	2 videos were recorded (nasal and temporal conjunctiva) Settings on 16 × and white diffuse light	BHVI grading scale [24]:
			Grade 1 (very slight hyperemia)
			Grade 2 (slight hyperemia)
			Grade 3 (moderate hyperemia)
			Grade 4 (severe hyperemia)
Meibography	Oculus Keratograph 5 M	Eversion of the lower eyelid and several images were taken MGLA analyzed with ImageJ	Pult et al. [26] Meiboscale:
			Grade 1 (< 25%)
			Grade 2 (25–50%)
			Grade 3 (50–75%)
			Grade 4 (> 75%)

*OSDI*—ocular surface disease index, *VDT*—video display terminals, *MG*—meibomian glands

### Meibometry

Meibometry was performed with the Meibometer MB 560 (Courage-Khazaka electronic GmbH, Cologne, Germany) [16–19]. This device is a photometer unit connected to a computer that shows the recorded data. To collect the meibomian secretion, the manufacturer`s protocol was followed by using a matt synthetic tap which is also provided by them. The matt synthetic tape was bent and brought in contact with the central portion of the eyelid margin for 10 s. The meibomian secretion sample collected on the matt synthetic tape was let to dry for 1 min before it was introduced into the meibometer for measurement. Average mean was calculated from three readings of the same tape on the photometry unit. Data was measured in Meibometer Units (MU) [16, 18].

### Ocular surface examination under slit lamp

An exhaustive ocular surface examination under a Topcon SL-D4 (TOPCON Corporation, Tokyo, Japan) slit lamp with a video camera DC-4 attached was performed on every participant. Videos of each participants’ ocular surface were recorded for the posterior analysis by the second masked observer. Ocular surface exploration examined the eyelid abnormalities, quality of the MG secretion and conjunctival hyperemia. Different settings were used on the slit lamp for the thorough examination of each ocular structure.

### Eyelid margin abnormalities

For the eyelid margin observation, white diffuse light and 16 × magnification were used on the slit lamp. A video of the lower eyelid was recorded, and the following parameters were analyzed: eyelid margin irregularity, hyperemia, and plugging of the MG orifices. Eyelid abnormalities were categorized using the Arita et al.[20] grading scale (Table 1).

### Meibomian gland secretion quality

**Fig. 2:**
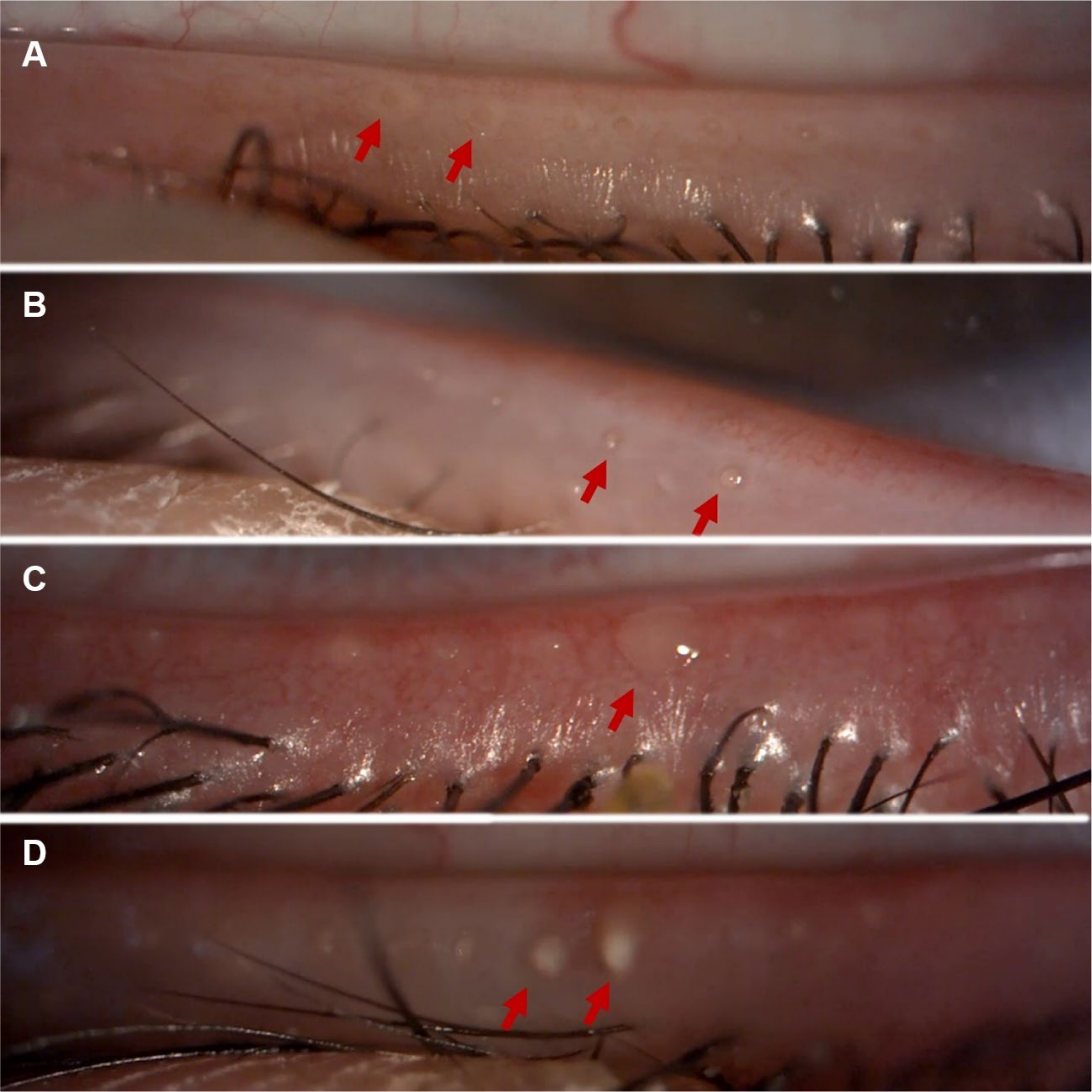
Classification of the MG secretion quality. A: grade 0 (clear); B: grade 1 (cloudy); C: grade 2 (granular); D: grade 3 (opaque solid/ toothpaste). *MG*—meibomian glands

To stimulate the MG secretion, digital pressure was applied for 30 s along the length of the lower eyelid where the MG are located [21]. For video capturing, 16 × and 25 × magnification, and diffuse white light were set on the slit lamp. A single video was recorded to capture the examination of the quality of the MG secretion of the lower eyelid. The quality of the MG secretion was analyzed following Bron et al.[22] grading scale (Table 1 and Fig. 2).

### Conjunctival hyperemia

A total of two videos of the bulbar conjunctiva were captured on the slit lamp with 16 × magnification and diffuse white light [23]. One video of the nasal and another of the temporal area of the bulbar conjunctiva. Videos were analyzed and categorized according to the Brien Holden Vision Institute (BHVI) grading scale [24] (Table 1).

### Meibography

Meibography images were captured by the Keratograph 5 M topographer (OCULUS Optikgeräte GmbH, Wetzlar, Germany) which uses an infrared camera for the MG in-vivo observation [25]. MGLA was measured by the difference between the total tarsus area and the MG area measured with the ImageJ open-source software (National Institutes of Health, Bethesda, Maryland, USA) [25–27]. Lower tarsus images were taken by everting lower eyelids while the participants were instructed to look to the ceiling. The MGLA data were graded according to the Meiboscale proposed by Pult et al.[26] (Table 1).

### Statistical analysis

IBM SPSS Statistics v.25 software (SPSS Inc., Illinois, USA) was used for the data statistical analysis. A significance value of *p* < 0.05 was used for all statistical tests. Data normality was checked using the Kolmogorov–Smirnov test; all parameters were not normally distributed (Kolmogorov–Smirnov, all *p* ≤ 0.001), except the meibometry (Kolmogorov–Smirnov, *p* = 0.188) [28, 29].

Due to the non-continuous distribution of the data, correlations between values were assessed by performing the Spearman Rho Correlation test [28]. Since data were categorical variables, Fisher’s exact test was performed to assess the association between them [28, 29]. Differences in meibometry between quality of the MG secretion groups were studied by a Kruskal–Wallis test, while differences by pairs were assessed using the Mann–Whitney *U* test [28]. Multivariate linear regression was performed on the studied parameters and expressed with the coefficients B and *p* value.

## Results

### Descriptive and demographic statistics of the sample

The present study analyzed a total sample of 120 participants with a mean age (± SD) of 22.5 ± 2.32 years, of whom 32 (26.7%) were male and 88 (73.3%) were female. OSDI questionnaire showed a median (Interquartile Range [IQR]) of 2 (1–3) and a distribution of 52 (43.3%) participants were grade 1 (Normal group), 36 (30.0%) grade 2 (Mild group), 20 (16.7%) grade 3 (Moderate group), and 12 (10.0%) grade 4 (Severe group). Hours of VDT use showed a median (IQR) of 2 (1–3) and a distribution of 40 (33.3%) participants were in the < 6 h/day group, 40 (33.3%) were in the 6–8 h/day group, and 40 (33.3%) were in the > 8 h/day group. Meibometry showed a mean (± SD) value of 283.88 ± 128.36 MU. Eyelid margin irregularity showed a median (IQR) of 0 (0–0) and a distribution of 100 (83.3%) participants were grade 0, and 20 (16.7%) grade 1. Eye lid margin hyperemia showed a median (IQR) of 1 (0–2) and a distribution of 41 (34.2%) participants were grade 0, 43 (35.8%) grade 1, 30 (25.0%) grade 2, and 6 (5.0%) grade 3. Plugging of the MG orifices showed a median (IQR) of 0 (0–1) and a distribution of 80 (66.7%) participants were grade 0, 30 (25.0%) grade 1, 6 (5.0%) grade 2, and 4 (3.3%) grade 3. Quality of the MG secretion showed a median (IQR) of 0 (0–1) and a distribution of 63 (52.5%) participants were grade 0, 46 (38.3%) grade 1, 7 (5.8%) grade 2, and 4 (3.3%) grade 3. Nasal conjunctival hyperemia showed a median (IQR) of 2 (2–3) and a distribution of 13 (10.8%) participants were grade 1, 67 (55.8%) grade 2, 37 (30.8%) grade 3, and 3 (2.5%) grade 4. Temporal conjunctival hyperemia showed a median (IQR) of 2 (2–3) and a distribution of 20 (16.7%) participants were grade 1, 69 (57.5%) grade 2, 29 (24.2%) grade 3, and 2 (1.7%) grade 4. MGLA showed a median (IQR) of 2 (2–2) and a distribution of 19 (15.8%) participants who were grade 1, 76 (63.3%) grade 3, 22 (18.3%) grade 3, and 3 (2.5%) grade 4.

### Correlations between studied parameters

Table 2 shows the correlations obtained between all the studied parameters. OSDI showed a statistically significant positive correlation with hours of VDT use (Spearman Rho test; *r* = 0.235, *p* = 0.010), and eyelid margin hyperemia (Spearman Rho test; *r* = 0.257, *p* = 0.005). A statistically significant correlation was found between the quality of the MG secretion, eyelid margin hyperemia (Spearman Rho test; *r* = 0.217, *p* = 0.017), plugging of the MG orifices (Spearman Rho test; *r* = 0.0372, *p* < 0.001), nasal conjunctival hyperemia (Spearman Rho test; *r* = 0.186, *p* = 0.042), temporal conjunctival hyperemia (Spearman Rho test; *r* = 0.217, *p* = 0.017), and MGLA (Spearman Rho test; *r* = 0.187, *p* = 0.041). Eyelid margin irregularity showed a statistical correlation with eyelid margin hyperemia (Spearman Rho test; *r* = 0.287, *p* = 0.001) between plugging of the MG orifices (Spearman Rho test; *r* = 0.248, *p* = 0.006). Also, plugging of the MG orifices was significantly correlated with nasal conjunctival hyperemia (Spearman Rho test; *r* = 0.314, *p* < 0.001), and temporal conjunctival hyperemia (Spearman Rho test; *r* = 0.383, *p* < 0.001).

**Table 2:** Correlations between all the studied parameters. *p* value were determined by Spearman Rho test

		OSDI	Hours of VDT use	Meibometry	Eyelid margin irregularity	Eyelid margin hyperemia	Plugging of the MG offices	Quality of the MG secretion	Nasal conjunctival hyperemia	Temporal conjunctival hyperemia
Hours of VDT use	*r*	0.235								
	*p*	0.010*								
Meibometry	*r*	− 0.074	0.034							
	*p*	0.424	0.711							
Eyelid margin irregularity	*r*	0.093	0.110	− 0.103						
	*p*	0.311	0.234	0.263						
Eyelid margin hyperemia	*r*	0.257	0.002	− 0.011	0.287					
	*p*	0.005*	0.984	0.908	0.001*					
Plugging of the MG offices	*r*	0.172	0.141	− 0.032	0.248	0.325				
	*p*	0.060	0.123	0.731	0.006	< 0.001*				
Quality of the MG secretion	*r*	0.085	0.104	− 0.170	0.141	0.217	0.372			
	*p*	0.356	0.261	0.063	0.124	0.017*	< 0.001*			
Nasal conjunctival hyperemia	*r*	0.098	− 0.046	0.056	0.181	− 0.080	0.314	0.186		
	*p*	0.285	0.616	0.547	0.048*	0.383	< 0.001*	0.042*		
Temporal conjunctival hyperemia	*r*	0.141	− 0.013	0.174	0.151	0.128	0.383	0.217	0.430	
	*p*	0.125	0.891	0.058	0.099	0.163	< 0.001*	0.017*	< 0.001*	
MGLA	*r*	− 0.002	− 0.023	− 0.152	0.079	0.099	0.058	0.187	0.058	0.092
	*p*	0.979	0.803	0.098	0.389	0.283	0.528	0.041*	0.528	0.315

^*^Statistically significant (*p* < 0.05)

*OSDI*—ocular surface disease index, *VDT*—video display terminals, *MG*—meibomian glands, *MGLA*—meibomian gland loss area

### Association between the DED symptomatology and studied parameters

Multivariate regression showed that the DED symptomatology was significantly associated with higher values of hours of VDT use (coefficients *B* = 0.316, *p* = 0.007) and higher values of eyelid margin hyperemia (coefficients *B* = 0.434, *p* ≤ 0.001). No other studied parameter showed any association (Table 3).

**Table 3:** Multivariate regression analysis performed for the OSDI score as the dependent variable and studied parameters as the covariables

	Coefficients		Standardized coefficients	*T*	*p*
	B	SD	β		
Constant	− 0.518	1.277	–	− 0.406	0.686
Age	0.034	0.044	0.078	0.766	0.446
Sex	0.434	0.236	0.192	1.835	0.069
Hours of VDT use	0.316	0.114	0.258	2.765	0.007*
Meibometry	− 0.001	0.001	− 0.132	− 1.415	0.160
Eyelid margin irregularity	− 0.236	0.260	− 0.088	− 0.905	0.367
Eyelid margin hyperemia	0.434	0.119	0.387	3.665	< 0.001*
Plugging of the MG orifices	− 0.109	0.151	− 0.081	− 0.723	0.471
Quality of the MG secretion	− 0.144	0.139	− 0.107	− 1.029	0.306
Nasal conjunctival hyperemia	0.170	0.153	0.115	1.111	0.269
Temporal conjunctival hyperemia	0.183	0.158	0.125	1.156	0.250
MGLA	0.001	0.143	< 0.001	0.004	0.997

Analysis was adjusted for age and sex

*SD*—standard deviation, *MG*—meibomian glands, *OSDI*—ocular surface disease index, *VDT*—video display terminals, *MGLA*—meibomian gland loss area

*p* values were determined by multivariate regression analysis

^*^Statistically significant (*p* < 0.05)

### Association between the gender and studied parameters

Table 4 shows the gender association among studied parameters, where the distribution of the female sample showed statistical association with the eyelid margin hyperemia (Fisher’s exact test; *p* < 0.001), MGLA (Fisher’s exact test; *p* = 0.015), nasal (Fisher’s exact test; *p* = 0.014) and temporal (Fisher’s exact test; *p* = 0.006) conjunctival hyperemia. No other parameter showed statistical association with the female or male gender.

**Table 4:** Distribution of the data according to gender and all the categorical studied parameters

Parameter	Category	Gender		Total	*p*
		Male	Female		
OSDI	Grade 1	14	38	52	0.904
	Grade 2	10	26	36	
	Grade 3	6	24	20	
	Grade 4	2	10	12	
Hours of VDT use	Grade 1	12	28	40	0.536
	Grade 2	12	28	40	
	Grade 3	8	32	40	
Eyelid margin irregularity	Grade 0	26	74	100	0.783
	Grade 1	6	14	20	
	Grade 2	0	0	0	
Eyelid margin hyperemia	Grade 0	8	31	41	< 0.001*
	Grade 1	7	36	43	
	Grade 2	11	19	30	
	Grade 3	6	0	6	
Plugging of the MG orifices	Grade 0	20	60	80	0.077
	Grade 1	9	21	30	
	Grade 2	0	6	6	
	Grade 3	3	1	4	
Quality of the MG secretion	Grade 0	16	47	63	0.435
	Grade 1	11	35	46	
	Grade 2	3	4	7	
	Grade 3	2	2	4	
MGLA	Grade 1	3	16	19	0.015*
	Grade 2	23	53	76	
	Grade 3	3	19	22	
	Grade 4	3	0	3	
Nasal conjunctival hyperemia	Grade 1	1	12	13	0.014*
	Grade 2	20	47	67	
	Grade 3	8	29	37	
	Grade 4	3	0	3	
Temporal conjunctival hyperemia	Grade 1	1	19	20	0.006*
	Grade 2	18	51	69	
	Grade 3	11	18	29	
	Grade 4	2	0	2	

*MG*—meibomian glands, *OSDI*—ocular surface disease index, *VDT*—video display terminals, *MGLA*—meibomian gland loss area

*p* values were determined by Fisher’s exact test

^*^Statistically significant (*p* < 0.05)

### Association and differences between the quality of the MG secretion and studied parameters

The mean ± SD values of meibometry for each MG secretion quality grade were measured as: grade 0 (299.83 ± 131.48 MU), grade 1 (280.93 ± 125.12 MU), grade 2 (203.81 ± 123.34 MU), and grade 3 (206.83 ± 51.38 MU). No statistical differences were found in meibometry values between the MG secretion quality grades (Kruskal–Wallis test; *p* = 0.125). Additionally, differences by pairs were performed and no statistical differences were found (Mann-Witney *U* test; all *p* ≥ 0.055).

The relationship between the quality of the MG secretion and OSDI, hours of VDT use, eyelid margin abnormalities, conjunctival hyperemia, and MGLA are reported as a contingency table (Table 5). No statistical relationship was found between the quality of the MG secretion and OSDI (Fisher’s exact test; *p* = 0.238), and hours of VDT use (Fisher’s exact test; *p* = 0.392). Although, a statistical association between eyelid margin hyperemia (Fisher’s exact test; *p* = 0.027) and plugging of the MG orifices (Fisher’s exact test; *p* < 0.001) was found; when these parameters vary the MG secretion quality grade varied too. No statistical relationship was found between the quality of the MG secretion and eyelid margin irregularity (Fisher’s exact test; *p* = 0.139). Contingency Table 5 shows significant variation in nasal and temporal conjunctival hyperemia among the different MG secretion quality grades, it occurs in both areas: nasal (Fisher’s exact test; *p* = 0.023), and temporal (Fisher’s exact test; *p* = 0.039). MGLA grade significantly varied as the quality of the MG secretion varied (Fisher’s exact test; *p* = 0.013).

**Table 5:** Distribution of the data according to the quality of the MG secretion and all the categorical studied parameters

Parameter	Category	Quality of the MG secretion				Total	*p*
		Grade 1	Grade 2	Grade 3	Grade 4		
OSDI	Grade 1	31	17	3	1	52	0.238
	Grade 2	15	16	2	3	36	
	Grade 3	13	5	2	0	20	
	Grade 4	4	8	0	0	12	
Hours of VDT use	Grade 1	25	13	2	0	40	0.392
	Grade 2	17	19	3	1	40	
	Grade 3	21	14	2	3	40	
Eyelid margin irregularity	Grade 0	55	38	4	3	100	0.139
	Grade 1	8	8	3	1	20	
	Grade 2	0	0	0	0	0	
Eyelid margin hyperemia	Grade 0	24	16	1	0	41	0.027*
	Grade 1	25	15	2	1	43	
	Grade 2	14	11	2	3	30	
	Grade 3	0	4	2	0	6	
Plugging of the MG orifices	Grade 0	51	26	2	1	80	< 0.001*
	Grade 1	10	16	4	0	30	
	Grade 2	2	3	0	1	6	
	Grade 3	0	1	1	2	4	
MGLA	Grade 1	12	6	1	0	19	0.013*
	Grade 2	41	30	3	2	76	
	Grade 3	10	10	2	0	22	
	Grade 4	0	0	1	2	3	
Nasal conjunctival hyperemia	Grade 1	8	5	0	0	13	0.023*
	Grade 2	39	21	5	2	67	
	Grade 3	16	19	0	2	37	
	Grade 4	0	1	2	0	3	
Temporal conjunctival hyperemia	Grade 1	12	8	0	0	20	0.039*
	Grade 2	40	23	4	2	69	
	Grade 3	11	15	2	1	29	
	Grade 4	0	0	1	1	2	

*MG*—meibomian glands, *OSDI*—ocular surface disease index, *VDT*—video display terminals, *MGLA*—meibomian gland loss area

*p* values were determined by Fisher’s exact test

^*^Statistically significant (*p* < 0.05)

Multivariate regression showed that the quality of the MG secretion was significantly associated with higher values of the MG orifice plugging (coefficients *B* = 0.295, *p* = 0.004) and lower values of meibometry (coefficients *B* = − 0.001, *p* = 0.029). No other studied parameter showed any association (Table 6).

**Table 6:** Multivariate regression analysis performed for quality of the MG secretion as the dependent variable and studied parameters as the covariables. Analysis was adjusted for age and sex

	Coefficients		Standardized coefficients	*T*	*p*
	B	SD	β		
Constant	− 0.140	0.878	–	− 0.159	0.874
Age	− 0.004	0.030	− 0.012	− 0.129	0.898
Sex	0.041	0.165	0.025	0.251	0.802
OSDI	− 0.068	0.066	− 0.091	− 1.029	0.306
Hours of VDT use	0.103	0.081	0.112	1.273	0.206
Meibometry	− 0.001	< 0.001	− 0.188	− 2.217	0.029*
Eyelid margin irregularity	− 0.076	0.179	− 0.038	− 0.424	0.672
Eyelid margin hyperemia	0.147	0.085	0.175	1.725	0.087
Plugging of the MG orifices	0.295	0.100	0.292	2.948	0.004*
Nasal conjunctival hyperemia	0.066	0.106	0.060	0.629	0.531
Temporal conjunctival hyperemia	0.140	0.109	0.128	1.288	0.200
MGLA	0.155	0.097	0.137	1.600	0.113

*SD*—standard deviation, *MG*—meibomian glands, *OSDI*—ocular surface disease index, *VDT*—video display terminals, *MGLA*—meibomian gland loss area

*p* values were determined by multivariate regression analysis

^*^Statistically significant (*p* < 0.05)

## Discussion

The number of hours using VDT by university students during the COVID-19 pandemic increased substantially and could influence their ocular surface health [2, 3]. In fact, the number of complete blinks during the use of VDT was measured to be reduced [4, 5]; incomplete blinking reduces the meibum spreading over the ocular surface, leading to the accumulation of fatty acids over the eyelids free border, and causing MG orifices obstruction with the subsequent reduction of the MG secretion quality [8].

Although the main objective of this research was to analyze if the quality of the MG secretion was associated with other ocular surface parameters, results showed a positive correlation between VDT use and ocular symptomatology as well as multivariate regression showed an influence of the hours of VDT use in the DED symptomatology. Similar results to the present study were observed by García-Ayuso et al.[30] who studied 676 university students and observed higher OSDI values due to synchronous hybrid learning during the COVID-19 pandemic. Also, other researchers performed only surveys in healthy university students during the COVID-19 pandemic and observed an increase in ocular discomfort consistent with DED symptomatology [3, 31, 32]. Fenga et al. [6] also observed differences in symptomatology values in a sample of participants that use VDT with and without MG dysfunction. Wu et al. [33] studied 93 participants with dry eye symptomatology distributed into two VDT users groups, a positive correlation between hours of VDT use and OSDI was found. Ocular discomfort could also be due to incomplete blinks during the use of VDT; this hypothesis was supported by Portello et al.[7] which found higher values of dry eye symptomatology in participants with lower blink rates and high ratios of incomplete blinks during different visual tasks.

A normal blink rate and complete blinking could be factors that do not allow the obstruction of the MG orifices by the meibum accumulated over the eyelid border, with the consequent alteration of the quality of the MG secretion [4]. The present study found positive correlations between the quality of the MG secretion and eyelid margin hyperemia, plugging of the MG orifices, and MGLA. Besides, Fisher’s exact test showed a relationship between eyelid margin hyperemia, plugging of the MG orifices, and MGLA when were distributed by grades of the MG secretion quality. All these results are corroborated by the multivariate regression analysis where plugging of the MG orifices was shown to cause a greater alteration in the MG secretion. These findings are in concordance with Wu et al.[33], who found significant differences between groups for eyelid margin abnormalities, quality of the MG secretion, and MGLA. Also, Zhou et al.[34] stated that lid margin score was a useful predictor for MG loss depending on the obstruction of the MG orifices. The relationship between MGLA, eyelid margin abnormalities, and the quality of the MG secretion must be considered [8], because incomplete blinking could accumulate lipids over the eyelid margin, which may change their chemical proprieties, and obstruct the MG orifices. A thicker MG secretion is more difficult to be spread over the ocular surface, and therefore cannot reach its anti-evaporative function which is the main purpose of the lipids secreted by the MGs. This finding could explain why the obstruction of the MGs orifices altered the quality of the MGs secretion and triggers the MGs destruction, which is in concordance with previous reports [34]. The use of VDT reduces the rate of complete blinks which may influence the quality of the MG secretion with the subsequent loss of ocular surface homeostasis [7].

Also, a significant relationship between nasal and temporal conjunctival hyperemia with quality of the MG secretion was found by both, Spearman Rho correlation and Fisher’s exact test. Participants with thicker MG secretion showed higher values of conjunctival hyperemia, which could be due to an inefficient evaporative function of the thicker meibum because of its difficulty to be spread over the ocular surface. To the authors’ knowledge, there are no other studies that evaluated conjunctival hyperemia and its relationship with the quality of the MG secretion and plugging of the MG orifices.

No differences were reported in meibometry values between the quality of the MG secretion grades. This could be because the meibometry technique only measures the quantity of lipids present on the matt synthetic tape and not their quality [16, 18]. It also does not consider the thickness of the meibum which depends of the chemical quality of the lipids (polar or not). Additionally, the authors noticed that MGs does not yield all at the same time point of the day and the present study was carried out from 10 am to 7 pm, not always at the same day hour [35], which could be a limitation of the study. Nevertheless, multivariate regression identified the meibometry as a causative factor for the alteration of the quality of the MG secretion, which could explain that lower meibometry values could be due to a worse quality of the MG secretion and higher values of plugging of the MG orifices.

## Conclusion

In conclusion, participants with higher values in the quality of the MG secretion have higher values in eyelid margin hyperemia, plugging of the MG orifices, MGLA, nasal conjunctival hyperemia, and temporal conjunctival hyperemia. No direct relationship between the quality of the MG secretion and hours of VDT use, or OSDI was found. Participants that spend long hours using VDT had higher values in OSDI, which can be explained by the impact of VDT in complete blinking, responsible for spreading the MG secretion over the ocular surface to avoid tear evaporation. To support the hypothesis proposed on how to complete blinking and VDT use affect the eyelid border and MGs, other parameters like blink rates, tear film break-up time, ocular protection index, and tear film lipid layer interference patterns should be measured in future research.
